# A Novel Halotolerant Thermoalkaliphilic Esterase from Marine Bacterium *Erythrobacter seohaensis* SW-135

**DOI:** 10.3389/fmicb.2017.02315

**Published:** 2017-11-22

**Authors:** Ying-Yi Huo, Zhen Rong, Shu-Ling Jian, Cao-Di Xu, Jixi Li, Xue-Wei Xu

**Affiliations:** ^1^Key Laboratory of Marine Ecosystem and Biogeochemistry, Second Institute of Oceanography, State Oceanic Administration, Hangzhou, China; ^2^State Key Laboratory of Genetic Engineering, Collaborative Innovation Center of Genetics and Development, School of Life Sciences, Shanghai Engineering Research Center of Industrial Microorganisms, Fudan University, Shanghai, China

**Keywords:** esterase, family IV, marine, alkaliphilic, halotolerant, homology modeling

## Abstract

A novel esterase gene, *e69*, was cloned from *Erythrobacter seohaensis* SW-135, which was isolated from a tidal flat sediment of the Yellow Sea in Korea. This gene is 825 bp in length and codes for a 29.54 kDa protein containing 274 amino acids. Phylogenetic analysis showed that E69 is a new member of the bacterial lipolytic enzyme family IV. This enzyme exhibited the highest level of activity toward *p*-nitrophenyl (NP) butyrate but little or no activity toward the other *p*-NP esters tested. The optimum temperature and pH of the catalytic activity of E69 were 60°C and pH 10.5, respectively. The enzyme exhibited stable activity over a wide range of alkaline pH values (7.5–9.5). In addition, E69 was found to be a halotolerant esterase as it exhibited the highest hydrolytic activity in the presence of 0.5 M NaCl and was still active in the presence of 3 M NaCl. Moreover, it possessed some degree of tolerance to Triton X-100 and several organic solvents. Through homology modeling and comparison with other esterases, it was suggested that the absence of the cap domain and its narrow substrate-binding pocket might be responsible for its narrow substrate specificity. Sequence and structural analysis results suggested that its high ratio of negatively to positively charged residues, large hydrophobic surface area, and negative electrostatic potential on the surface may be responsible for its alkaline adaptation. The results of this study provide insight into marine alkaliphilic esterases, and the unique properties of E69 make it a promising candidate as a biocatalyst for industrial applications.

## Introduction

Esterases (EC 3.1.1.1) are lipolytic enzymes that catalyze the cleavage of ester bonds of short-chain fatty esters in aqueous media and the reverse reaction in organic solvents and are widely expressed in various organisms, including bacteria, fungi, vertebrates, and invertebrates (Arpigny and Jaeger, 1999; Fuciños et al., 2012; Lópezlópez et al., 2014). Esterases have diverse industrial applications because of their broad substrate specificity, stereoselectivity, no requirements for cofactors, and high stability in organic solvents (Bornscheuer, 2002; Sayali and Surekha, 2013). They have wide applications as biocatalysis in food, detergent, textile, paper, pharmaceutical and agrochemical industries, synthesis of biopolymers, biodiesel production, and bioremediation and waste treatment (Barone et al., 2014; Lópezlópez et al., 2014; Moreno et al., 2016; Parte et al., 2017).

Marine environments are extremely diverse and marine microbial enzymes are of increasing interest because of their ability to withstand harsh conditions, including high or low temperatures, high or low pH, high salt concentrations, and high pressure (Demirjian et al., 2001; Trincone, 2011). Moreover, since high salt concentration tends to reduce the water activity like organic solvents, esterases from marine microorganisms have potentials in industrial biocatalytic processes in the presence of organic solvents, high salinity, and low water activity environments, such as stereospecific reaction, esterification, transesterification, and polymerization reaction (Fuciños et al., 2012). Recently, an increasing number of esterases with habitat-specific characteristics have been identified from marine environments or marine microorganisms, including esterases that are thermostable (Li et al., 2015; Huang et al., 2016), cold-active (Fu et al., 2011; Jiang et al., 2012b; De Santi et al., 2014; Tchigvintsev et al., 2015), alkaliphilic (Park et al., 2007; De Santi et al., 2014), halotolerant (Jiang et al., 2012a; Fang et al., 2014; De Santi et al., 2016a; Zhang et al., 2017), or tolerant to solvents (Zhang et al., 2014; Guo et al., 2016).

Bacterial lipolytic enzymes were initially classified into eight families (I–VIII) according to their amino acid sequences, and nine additional families (IX–XVII) were subsequently added (Arpigny and Jaeger, 1999; Lenfant et al., 2013; Castilla et al., 2017). Family IV esterases share remarkable amino acid sequence similarity with mammalian hormone-sensitive lipase (HSL) and have also been referred to as the HSL family (Arpigny and Jaeger, 1999). Many members of this family have been characterized as thermoactive and thermostable esterases (Hotta et al., 2002; Li et al., 2014, 2015; Huang et al., 2016). Nevertheless, only a few of these have been found to be alkaliphilic, especially with an optimum pH over 9.0. These include EstJ, which was cloned from a soil metagenome and exhibits its highest activity at pH 9.5 (Choi et al., 2013), and 499EST from *Acidicaldus* sp. strain USBA-GBX-499, which was isolated from a hot spring and exhibits its highest activity at pH 9.0 (López et al., 2014). To the best of our knowledge, a highly alkaliphilic family IV esterase from the marine environment has not been reported until now.

In this study, a novel esterase gene (*e69*) was cloned from *Erythrobacter seohaensis* SW-135, which was isolated from a tidal flat of the Yellow Sea (Yoon et al., 2005), and heterologously expressed. Sequence analysis suggested that E69 belongs to the bacterial lipolytic family IV. This enzyme was found to be highly alkaliphilic, thermophilic, halotolerant, and resistant to detergents and organic solvents. These characteristics indicate that E69 may be a promising candidate for various industrial applications.

## Materials and Methods

### Strains, Vectors, and Chemicals

*Erythrobacter seohaensis* SW-135 was kindly provided by Prof. Jung-Hoon Yoon and cultivated in marine broth 2216 (BD Difco^TM^, United States) at 30°C (Yoon et al., 2005). Kits for genomic DNA isolation, DNA purification, and plasmid isolation were purchased from Omega (United States). DNA polymerase was purchased from TaKaRa (China) and T4 DNA ligase and restriction endonucleases were purchased from New England Biolabs (United States). Plasmid pSMT3 (Herrmann et al., 1996) was stored in our lab and used as the vector for gene cloning and sequencing as well as protein expression. *Escherichia coli* BL21 (DE3) was used for protein expression. *E. coli* strains were grown at 37°C in Lysogeny broth (LB) medium containing 10 g/L NaCl, 10 g/L tryptone, and 5 g/L yeast extract (BD Difco^TM^, United States), pH 7.0, supplemented with kanamycin (50 μg/mL) when required. Ni Sepharose (GE Healthcare, United States) was used to purify the His_6_-tagged protein. *p*-Nitrophenyl (NP) acetate (C2), *p*-NP butyrate (C4), *p*-NP caprylate (C8), *p*-NP decanoate (C10), *p*-NP laurate (C12), *p*-NP myristate (C14), and *p*-NP palmitate (C16) were purchased from Sigma–Aldrich (United States) and *p*-NP hexanoate (C6) was purchased from TCI (Japan).

### Screening and Sequence Analysis of the Esterase Gene

The genome of *E. seohaensis* SW-135 was sequenced and annotated using high-throughput technologies (data not shown). A putative esterase gene designated *e69* was screened and the deduced amino acid sequence was analyzed using the blastp program^1^ (Altschul et al., 1990). Multiple sequence alignment of the amino acid sequences was performed using Clustal X version 2 (Larkin et al., 2007) and ESPript 3.0 (Robert and Gouet, 2014). The phylogenetic tree was constructed using the neighbor-joining method (Saitou and Nei, 1987) using the MEGA version 7.0 software (Kumar et al., 2016). The total amino acid compositions were calculated online using the ProtParam tool^2^.

### Cloning, Expression, and Purification of E69

The full-length gene of *e69* was amplified by PCR using the primers 5′-TCGCGGATCCATGCGTGACCACGGC-3′ (*Bam*HI site underlined) and 5′-TCCGCTCGAGTCAGTCTTCCTTCGCTG-3′ (*Xho*I site underlined), and cloned into the *Bam*HI–*Xho*I site of the expression vector pSMT3 to produce the N-terminal His-tagged small ubiquitin-related modifier (SUMO) fusion. The recombinant plasmid (pSMT3-*e69*) was transformed into *E. coli* BL21 (DE3) cells. Transformants harboring the recombinant plasmid were identified by PCR and further confirmed by DNA sequencing. The cells were cultivated at 37°C and 200 rpm until the optical density (OD_600_) reached approximately 0.6. The induction was then initiated by adding 1 mM of isopropyl-β-D-thiogalactopyranoside (IPTG) at 20°C and 200 rpm. After cultivation for 12 h, cells were collected by centrifugation at 12,000 rpm and 4°C for 10 min and then washed with phosphate-buffered saline (0.8% NaCl, 0.02% KCl, 0.142% Na_2_HPO_4_, 0.027% KH_2_PO_4_, pH 7.4). The cells were suspended in 20 mM imidazole buffer (500 mM NaCl, 20 mM Tris–HCl, pH 8.0) and subjected to ultrasonic disruption (350 W). After centrifugation at 12,000 rpm and 4°C for 30 min, the supernatant was purified with Ni Sepharose. Subsequently, the fusion protein was cleaved with ubiquitin-like specific protease 1 (ULP1) with overnight dialysis at 4°C. The digestion products were passed through the Ni Sepharose column to capture the His-tagged SUMO. The recombinant protein in the eluate was tested by sodium dodecyl sulfate–polyacrylamide gel electrophoresis (SDS–PAGE) using 12% polyacrylamide gels.

### Enzyme Activity Assay

The esterase activity assay was performed using a spectrophotometric method. The standard reaction mixture contained 10 μL of 100 mM *p*-NP butyrate, 980 μL of *N*-cyclohexyl-2-aminoethanesulfonic acid (CHES) buffer (50 mM, pH 10.0), and the purified enzyme in a final volume of 1 mL. The absorbance at 405 nm was monitored at 40°C and over 2 min using a DU800 ultraviolet–visible spectrophotometer (Beckman Coulter, Inc., United States). All values were determined in triplicate, and reactions to which thermally inactivated enzyme had been added were used as controls. One unit of enzyme activity was defined as the amount of esterase required to release 1 μmol of *p*-nitrophenol per minute from the *p*-NP ester.

### Enzyme Characterization

The substrate specificity was determined using *p*-NP esters with various acyl chain lengths (C2–C16), which were added into the reaction mixture to a final concentration of 1 mM.

The kinetic parameters were obtained using *p*-NP butyrate as the substrate at various concentrations (0.05, 0.2, 0.4, 0.6, 0.8, 1.0, 1.5, 1.8, 2.0, 2.5, 3.0, and 4.0 mM). The kinetic parameters were calculated by analyzing the slopes of the Michaelis–Menten equation using the GraphPad software (GraphPad Software, Inc., United States).

The optimum temperature for enzyme activity was determined over the range of 15–70°C. To study the thermostability, the residual activity of the enzyme was measured after incubation at various temperatures (30, 40, 50, and 60°C) for 1 or 2 h. The activity of the enzyme without treatment was defined as 100%.

The optimum pH for enzyme activity was determined over the pH range from 4.0 to 10.5. The liberated *p*-nitrophenol was measured at 348 nm, the pH-independent isosbestic wavelength of *p*-nitrophenol and *p*-nitrophenolate. The stability of E69 at various pH values was determined by incubating the enzyme in various buffers (pH 4.0–10.5) at 30°C for 2 h and measuring the remaining activity. The buffers used were 100 mM citrate buffer (pH 4.0–6.0), 100 mM phosphate buffer (pH 6.0–7.5), 100 mM tricine buffer (pH 7.5–9.0), and 50 mM CHES buffer (pH 9.0–10.5).

The effect of NaCl was determined by adding various concentrations of NaCl (0, 0.5, 1, 2, 3, 4, and 5 M) to the assay mixture. The enzyme activity without any addition of NaCl was defined as 100%. The effect of the chelating agent ethylenediaminetetraacetic acid (EDTA) was evaluated at a final concentration of 10 mM. The effects of various metal ions (Ba^2+^, Ca^2+^, Mg^2+^, and Sr^2+^) were examined at a final concentration of 10 mM. The effects of various detergents were determined using SDS, Triton X-100, Tween-20, and Tween-80 at final concentrations of 1% and 5% (v/v, except w/v for SDS). The effects of various organic solvents were measured using acetone, acetonitrile, ethanol, *N*,*N*-dimethylformamide (DMF), dimethyl sulfoxide (DMSO), glycerol, isopropanol, and methanol at final concentrations of 5% and 15% (v/v).

All values were determined in triplicate. Data were presented as mean ± SD. Statistical analyses were performed with Student’s *t*-tests. Two-tailed unpaired Student’s *t*-tests were used to compare two groups. *p*-value less than 0.05 was considered statistically significant.

### Homology Modeling and Putative Structure Analysis

The three-dimensional (3D) structure of E69 was modeled using the Phyre2 server (Protein Homology/analogY Recognition Engine version 2.0^3^) (Kelley et al., 2015). Solvent-accessible surfaces were calculated using the CCP4 software suite (Cowtan, 2011). In all calculations, water molecules were excluded from the coordinate files. The surface electrostatic potentials of protein structures were calculated via PyMOL with the assistance of the Adaptive Poisson–Boltzmann Solver (APBS) plugin (Baker et al., 2001). Structural figures were created using the PyMOL software^4^.

### Nucleotide Sequence Accession Number

The nucleotide sequence of the esterase gene *e69* was deposited into the GenBank database under the accession number MF095674.

## Results

### Sequence Analysis of E69

A putative esterase gene of 825 bp, encoding a protein of 274 amino acids, was identified from the genome sequence of *E. seohaensis* SW-135 and named *e69*. The deduced amino acid sequence of the putative esterase gene was compared with known esterase amino acid sequences available from the GenBank nr database and the Protein Data Bank (PDB) database, which revealed relatively low identities with known esterases. E69 shared 42% sequence identity (107/254) with an esterase from *Burkholderia cepacia* (GenBank Accession No. WP_059620138), 39% identity (100/258) with an esterase from *Methylobacterium* sp. GXS13 (WP_058193004), 38% identity (100/266) with an esterase from *Budvicia aquatica* (WP_051323122), and 38% identity (100/263) with an esterase from *Bradyrhizobium stylosanthis* (WP_063685245) in the GenBank nr database, as well as 33% identity (88/264) with esterase E25 from sediment of the South China Sea (4Q05) (Li et al., 2014), 33% identity (83/253) with esterase MGS-MT1 from seawater of the Matapan basin (4Q3O) (Alcaide et al., 2015), and 31% identity (86/275) with esterase EstE5 from soil (3FAK) (Nam et al., 2009b) in the PDB database.

**FIGURE 1:**
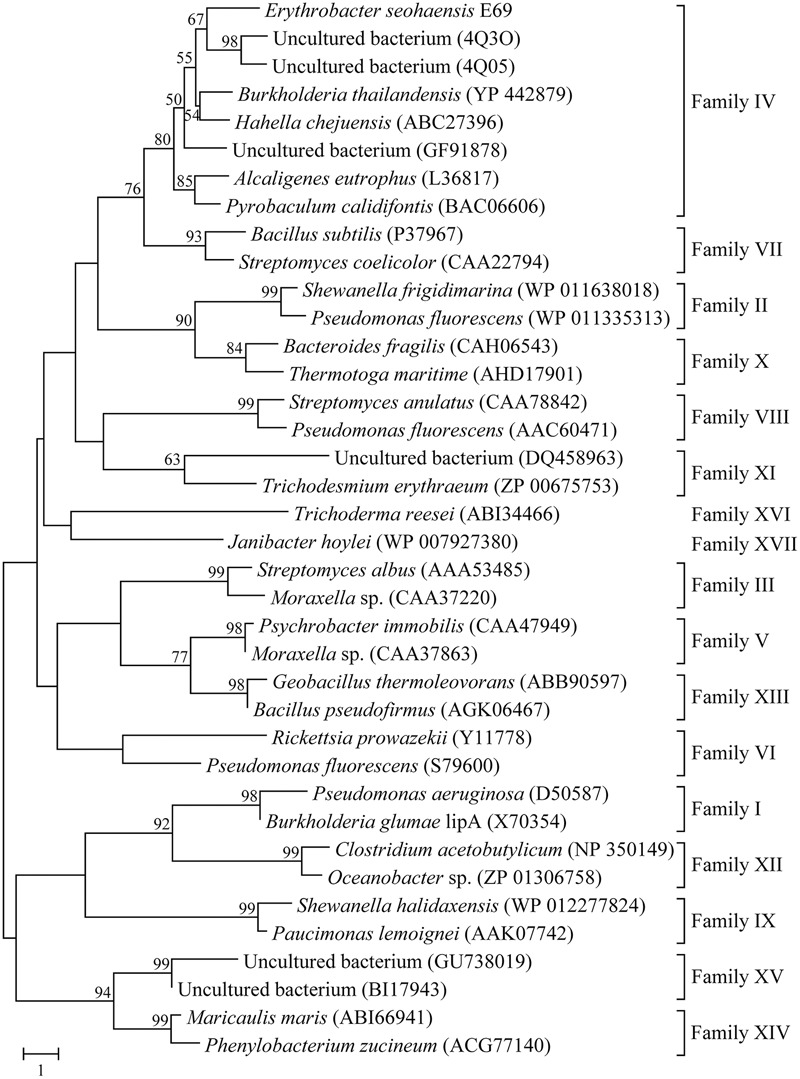
Neighbor-joining phylogenetic tree based on amino acid sequences of E69 and related lipolytic enzymes. Sequence alignment was performed using ClustalX and the tree was constructed using the MEGA software. Bootstrap values are based on 1000 replicates and only values of >50% are shown. The scale bar indicates the number of amino acid substitutions per site.

**FIGURE 2:**
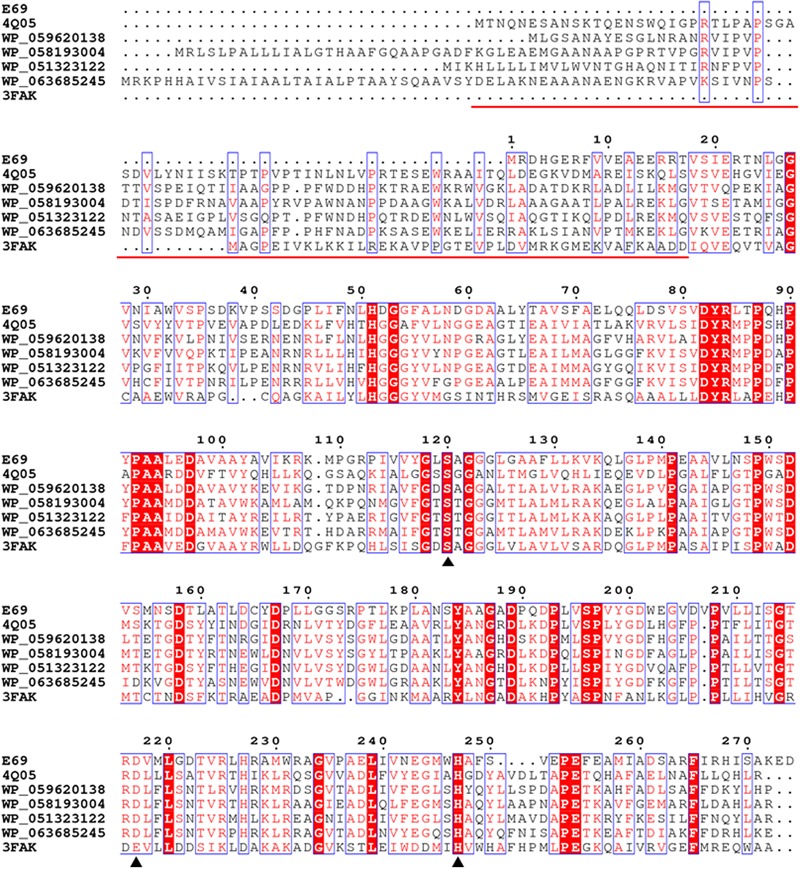
Amino acid sequence alignment of E69 and related lipolytic enzymes. Accession numbers of the enzymes in the PDB or GenBank databases are given. Sequence alignment was performed using the ClustalX and ESPript programs. Identical and similar residues among groups are shown in white text on a red background and in red text on a white background, respectively. The triangles indicate the locations of the catalytic active site residues [serine (S), aspartate (D), and histidine (H)]. The red line indicates the sequences of the cap domain in E25 (PDB: 4Q05) and EstE5 (PDB: 3FAK).

Phylogenetic analysis was carried out to reveal the relationship between E69 and other known bacterial lipolytic enzymes. The phylogenetic tree constructed with 17 bacterial lipolytic enzyme families showed that E69 belongs to family IV (**Figure 1**). The multiple sequence alignment of E69 with the most related homologs indicated that the catalytic triad of E69 is composed of Ser120, Asp217, and His247 residues (**Figure 2**). The catalytic residue Ser120 was located in the consensus pentapeptide sequence GXSXG (GLSAG).

### Expression and Purification of E69

The gene fragment coding for the enzyme was cloned into the pSMT3 vector and expressed in *E. coli* BL21 (DE3). After induction for 12 h with 1 mM IPTG at 20°C, the heterologously expressed protein was purified using His-tag affinity chromatography. The co-expressed His-tagged SUMO was then cleaved by ULP1 and removed by His-tag affinity chromatography. SDS–PAGE analysis of the purified protein revealed an approximate molecular weight (MW) of 30 kDa, which was consistent with the calculated value (29.54 kDa) (Supplementary Figure 1).

### Enzyme Characterization

**FIGURE 3:**
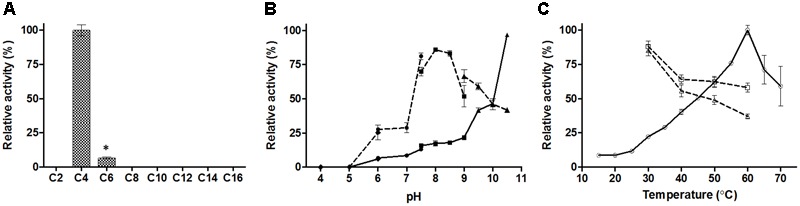
Activity of E69 toward various substrates **(A)**, and effects of pH **(B)** and temperature **(C)** on the activity and stability of E69. **(A)** Substrate specificity was determined using the *p*-NP esters. The highest activity was taken as 100%. ^∗^*p* < 0.05, representing a significant difference from the 100% values (Student’s *t*-test). **(B)** Activities at various pH values (solid lines) were assayed at 40°C, with the activity obtained at pH 10.5 taken as 100%. The residual activities (dashed lines) after incubation at various pH values for 2 h were assayed at 40°C and pH 10.0, and the value obtained without treatment was taken as 100%. Assay was performed in different buffers: 100 mM citrate buffer (pH 4.0–6.0, filled diamonds), 100 mM phosphate buffer (pH 6.0–7.5, filled circles), 100 mM tricine buffer (pH 7.5–9.0, filled squares), and 50 mM CHES buffer (pH 9.0–10.5, filled triangles). **(C)** Activities at various temperatures (solid line with empty circles) were assayed at pH 10.0 and the value obtained at 60°C was taken as 100%. The residual activities after incubation at various temperatures for 1 h (dashed line with empty squares) or 2 h (dashed line with empty triangles) were assayed at 40°C and pH 10.0, and the value obtained without treatment was taken as 100%. Data are presented as mean ± SD (*n* = 3).

The substrate specificity of E69 was determined using *p*-NP esters with various acyl chain lengths (C2–C16) (**Figure 3A**). Among the esters tested, E69 exhibited the highest activity toward *p*-NP butyrate (16.0 ± 1.1 U/mg) and only weak activity toward *p*-NP hexanoate (1.0 ± 0.1 U/mg). No activity was detected toward *p*-NP acetate or *p*-NP esters with side chains longer than C6. The *V*_max_ and *K*_m_ values of E69 were 34.9 ± 2.9 μmol/mg/min and 2.2 ± 0.3 mM, respectively, using *p*-NP butyrate as substrate.

The optimum activity of E69 was measured over a pH range of 4.0–10.5 and a temperature range of 15–70°C, with *p*-NP butyrate as the substrate. E69 showed the highest activity at pH 10.5 and 60°C (**Figures 3B,C**). The activity at pH values higher than 10.5 could not be tested, because of the high rate of self-degradation of *p*-NP butyrate. The pH stability and thermostability of E69 were determined by measuring the residual activity after incubation at various pH values and temperatures. The results showed that E69 was relatively stable over the alkaline pH range (7.5–9.5), retaining more than 50% of its initial activity after incubation for 2 h (**Figure 3B**). The thermostability analysis showed that E69 retained about 58–88% of its initial activity after incubation at 30–60°C for 1 h and 37–85% of its initial activity after 2 h (**Figure 3C**).

**FIGURE 4:**
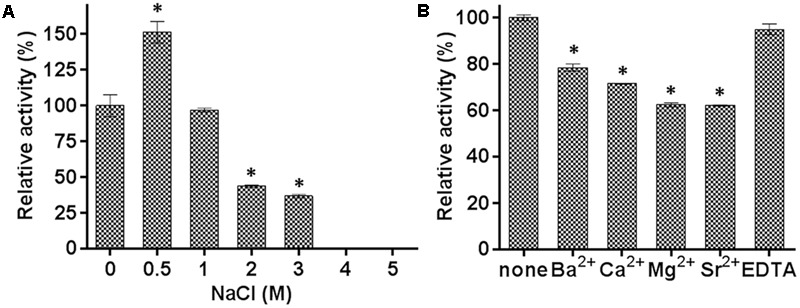
Effects of NaCl and metal ions on the activity of E69. **(A)** The effect of NaCl on the activity of E69 was determined using *p*-NP butyrate as the substrate. The value obtained without added NaCl was taken as 100%. **(B)** The effects of metal ions and EDTA on the activity of E69 were determined using *p*-NP butyrate as the substrate. All of the tests were performed at 40°C and pH 10.0. The value obtained without metal ions was taken as 100%. Data are presented as mean ± SD (*n* = 3). ^∗^*p* < 0.05, representing a significant difference from the 100% values (Student’s *t*-test).

The effects of various concentrations of NaCl and a range of metal ions, detergents, and organic solvents were also determined. The hydrolysis activity increased by about 50% in the presence of 0.5 M NaCl, and decreased to 95, 40, and 35% in the presence of 1.0, 2.0, and 3.0 M NaCl, respectively (**Figure 4A**). These results indicated that E69 was a halotolerant esterase, which might make it well adapted to the marine environment. The addition of 10 mM of Ba^2+^, Ca^2+^, Mg^2+^, or Sr^2+^ inhibited the activities of E69 (**Figure 4B**). E69 was highly resistant to acetonitrile, ethanol, DMF, glycerol, and isopropanol at concentrations of 5% (v/v), retaining over 70% of its initial activity (**Table 1**). However, the activity was strongly inhibited when the solvent concentrations were increased to 15% (v/v). The hydrolysis activity of E69 increased by about 65% and decreased slightly in the presence of 1 and 5% (v/v) of Triton X-100, respectively. Moreover, E69 still retained about 52, 28, and 20% of its activity in the presence of 1% of Tween 20 (v/v), Tween 80 (v/v), or SDS (w/v) (**Table 1**).

**Table 1:** Effects of various organic solvents and detergents on the activity of E69.

Solvent	Relative activity (%)		Detergent	Relative activity (%)	
	5%	15%		1%	5%
Control	100.0 ± 3.0		Control	100.0 ± 0.5	
Acetone	59.6 ± 3.6^∗^	8.9 ± 1.5^∗^	SDS	20.1 ± 0.2^∗^	0^∗^
Acetonitrile	85.4 ± 3.2^∗^	35.0 ± 4.0^∗^	Triton X-100	165.5 ± 3.9^∗^	74.3 ± 4.3^∗^
Ethanol	90.3 ± 0.1^∗^	13.9 ± 5.3^∗^	Tween 20	51.9 ± 3.3^∗^	0^∗^
DMF	74.8 ± 1.7^∗^	17.1 ± 4.1^∗^	Tween 80	27.9 ± 1.1^∗^	0^∗^
DMSO	42.1 ± 2.4^∗^	30.3 ± 0.3^∗^			
Glycerol	121.7 ± 14.4	0^∗^			
Isopropanol	91.3 ± 1.6^∗^	28.8 ± 1.3^∗^			
Methanol	0^∗^	0^∗^			

*Activities were determined using *p*-NP butyrate as the substrate at 40°C and pH 10.0. The activity without organic solvent or detergent was taken as 100%. ^∗^*p* < 0.05, representing a significant difference from the control (Student’s *t*-test).*

### Structural Modeling of E69

**FIGURE 5:**
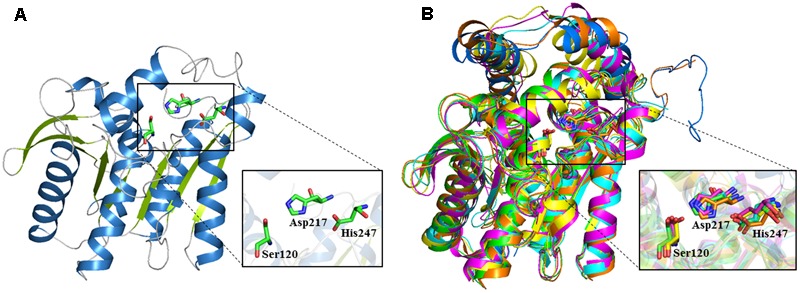
3D structure model of E69 and structural superimposition with other homologous esterases. **(A)** Cartoon representation of E69. The α helices and β strands are colored in blue and green, respectively. The catalytic triad residues are indicated as stick models colored in green. **(B)** The structural superposition of E69 (green), E25 (blue, PDB: 4Q05), MGS-MT1 (orange, PDB: 4Q3O), EstE5 (cyan, PDB: 3FAK), EstE7 (magenta, PDB: 3DNM), and E40 (yellow, PDB: 4XVC). The catalytic triad residues are indicated as stick models.

The 3D structure of E69 was modeled using the Phyre2 server. The structure showed a typical α/β-hydrolase folding as found for other esterases, in which parallel β strands were surrounded by several α helices (Holmquist, 2000) (**Figure 5A**). The structural model of E69 superimposed well with most related homologs, including E25 (Li et al., 2014), MGS-MT1 (Alcaide et al., 2015), EstE5 (Nam et al., 2009b), EstE7 (Nam et al., 2009a), and E40 (Li et al., 2015) (**Figure 5B**), and all of these esterases were isolated from metagenomics libraries. The predicted catalytic triad residues of E69, Ser120, Asp217, and His247, were indeed located in close proximity, and also superimposed well with those of most related homologs (**Figure 5B**).

**FIGURE 6:**
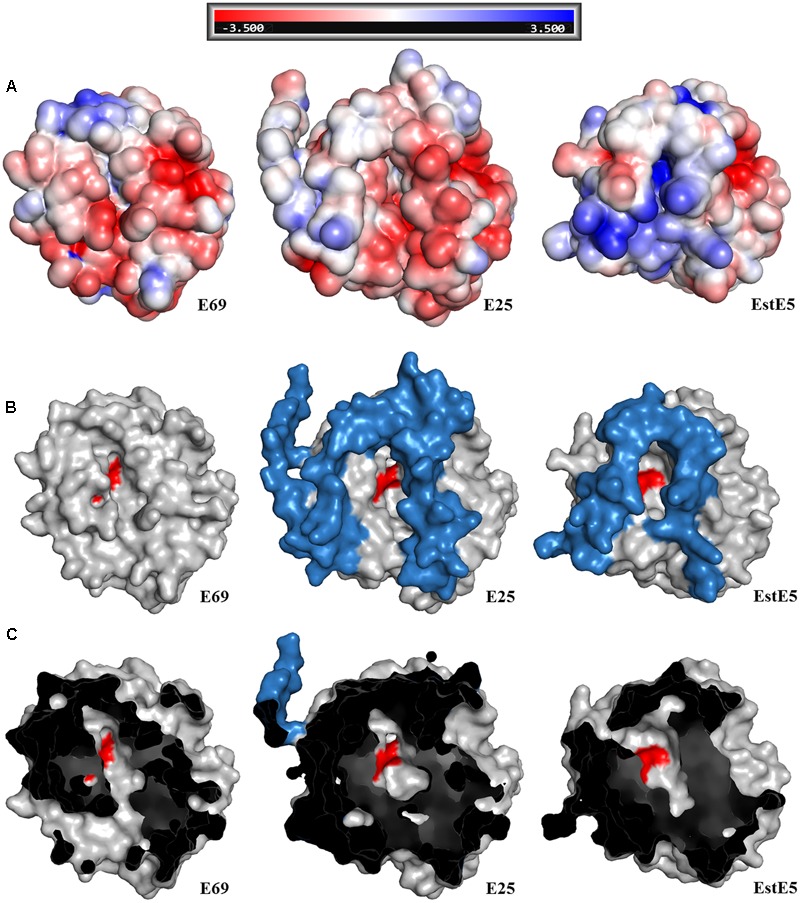
Structural comparison of E69 and its homologs, E25 (PDB: 4Q05) and EstE5 (PDB: 3FAK). **(A)** Electrostatic surface representations of E69 and its homologs, from -3.5 (red) to +3.5 kT/e (blue). Surface view of the structures showing the differences in **(B)** the cap domains and **(C)** the substrate-binding pockets. The cap domains and active sites are indicated in blue and red, respectively.

In order to understand the alkaline adaptation mechanisms of E69, 3D structural model of E69 was compared with those of the most closely related homologous proteins, whose optimum pH activities were not that high (7.0–8.5) (**Table 2**). The amino acid composition, solvent-exposed amino acids, and surface electrostatic potential of these esterases were analyzed (**Table 2**). Several characteristics contributing to the adaptation of E69 to the alkaline environment could be observed (**Table 2** and **Figure 6A**). Firstly, E69 possessed the fewest positively charged residues (22 for E69 and 25–31 for the others) and the highest ratio of negatively to positively charged residues (1.64 for E69 and 1.10–1.58 for the others) compared with the related esterases (**Table 2**). Secondly, analysis of the solvent-exposed residues revealed the highest ratio of surface hydrophobic residues to total hydrophobic residues (0.26 for E69 and 0.16–0.26 for the others) and the highest surface hydrophobic area for E69 (2635 Å^2^, accounting for 0.24 of the total hydrophobic area, compared with 1516–2294 Å^2^, accounting for 0.14–0.19, for the others) among these esterases, although its total numbers of hydrophobic residues and surface hydrophobic residues were not the highest (**Table 2**). Thirdly, the surfaces of the proteins with relatively high optimum pH, E69 and E25 (pH 10.5 and 8.5, respectively), were highly negatively charged, compared with those with near-neutral optimum pH (**Figure 6A**). All of these characteristics might lead to the increased stability of E69 and be responsible for its alkaline adaptation.

**Table 2:** Structural properties of E69 and its closely related alkaline esterases.

Feature	E69	E25^#^	MGS-MT1^#^	EstE5^#^	EstE7^#^	E40^#^
Accession number		4Q05	4Q3O	3FAK	3DNM	4XVC
Strain	*E. seohaensis* SW-135	Uncultured	Uncultured	Uncultured	Uncultured	Uncultured
Identity with E69 (%)	–	33	33	31	35	32
**Enzymatic characteristics**				
Optimum pH	>10.5	8.5	8	7.5	7.0	8.0
Optimum temperature (°C)	60	50	40	35	40	45
Optimum substrate	C4	C4	–	C4	C4	C4
**Amino acid composition**				
Total residues	274	340	348	297	309	297
Number of negatively charged residues^a^	36	39	39	34	41	37
Number of positively charged residues^a^	22	25	25	31	26	28
Charged residue ratio (negative/positive)	1.64	1.56	1.56	1.10	1.58	1.32
Total number of hydrophobic residues	120	138	134	133	125	132
**Surface amino acids with solvent accessibility of ≥30%**				
Total residues	94	95	112	112	101	101
Number of hydrophobic residues^b^	31	22	29	35	23	22
Ratio of surface hydrophobic residues/total hydrophobic residues	0.26	0.16	0.22	0.26	0.18	0.17
**Surface area of amino acids with solvent accessibility of ≥30%**				
Total surface area (Å^2^)	11,177	12,014	13,131	12,345	11,186	11,229
Surface hydrophobic area (Å^2^)	2635	1669	1985	2294	1553	1516
Ratio of surface hydrophobic area/total hydrophobic area	0.24	0.14	0.15	0.19	0.14	0.14

*^#^Sequence and structural informations were calculated based on E25 (Li et al., 2014), MGS-MT1 (Alcaide et al., 2015), EstE5 (Nam et al., 2009b), EstE7 (Nam et al., 2009a), and E40 (Li et al., 2015). ^a^The negatively charged residues were Asp and Glu; the positively charged residues were Arg and Lys. ^b^The hydrophobic residues were Ala, Ile, Leu, Pro, Trp, and Val.*

## Discussion

Marine enzymes, which are characterized by habitat-related features such as salt and pH tolerance, thermostability, barophilicity, adaptation to cold, and novel chemical and stereochemical properties, are of increasing interest due to their potential application as biocatalysts in industry (Trincone, 2011). The data mining of bacterial genomes for the screening and identification of enzyme-encoding genes has become an effective way to hunt for novel biocatalysts such as esterases, as a result of the ongoing development of sequencing technologies. Several novel marine esterases with diverse enzymatic properties have been screened out using a genome-sequence-based approach (Jiang et al., 2012a; Wei et al., 2013; De Santi et al., 2014, 2016b). Likewise, with the assistance of this approach, we identified the novel esterase E69 from the marine bacteria *E. seohaensis* SW-135.

E69 exhibits a narrow substrate specificity (**Figure 3A**). However, its most closely related family IV homologs, E25 and EstE5, demonstrated a much broader substrate specificity toward *p*-NP esters (C2–C10) (Nam et al., 2009b; Li et al., 2014). Comparisons of the structural models of E69, E25, and EstE5 revealed that E69 did not have a complete cap domain covering the substrate-binding pocket (**Figures 2, 6B**). The cap domain, which consists of approximately 50 amino acid residues, is commonly observed in family IV esterases and is considered to play an important role in enzyme activity, stability, and specificity (Mandrich et al., 2005; Li et al., 2014). Moreover, E69 possesses a relatively narrow substrate-binding pocket (**Figure 6C**), which may increase steric repulsion with the substrates and thus affect the substrate accommodation and limit the size of potential substrates. In addition, the activity of E69 toward other substrates was determined, including coenzyme A esters, α-naphthyl ester and β-naphthyl ester, and no activity was found (data not shown). In summary, the lack of a cap domain and the narrow substrate-binding pocket may together contribute to the observed narrow substrate specificity of E69.

Lipolytic enzymes that are functional under extreme conditions (e.g., high or low temperatures, high salinity, and high or low pH) have great application potential in the food, detergent, textile, and pharmaceutical industries (Gomes and Steiner, 2004; Fuciños et al., 2012). Only a few family IV esterases have been found to be alkaliphilic, including EstKT4 and EstKT9 from a tidal flat sediment metagenome (highest activity at pH 8.5) (Jeon et al., 2012), EstJ from a soil metagenome (highest activity at pH 9.5) (Choi et al., 2013), and 499EST from *Acidicaldus* sp. strain USBA-GBX-499 (highest activity at pH 9.0) (López et al., 2014). E69 showed the highest hydrolytic activity toward *p*-NP butyrate at pH 10.5 over the tested pH range of 4.0–10.5. However, the activity at higher pH values could not be determined, because of the high self-degradation rate of the substrate. To the best of our knowledge, E69 has the highest optimum pH of all of the reported family IV esterases. Moreover, it was stable over a wide range of alkaline pH (**Figure 3B**).

According to previous studies, enzymes that function under alkaline conditions employ several adaptation mechanisms, including but not limited to more negatively charged residues, more negatively charged solvent-exposed residues, a highly negatively charged surface, a large hydrophobic surface area, more salt bridges, and more salt bridges formed by arginine, which was determined based on the analysis of alkaline amylase, protease, and xylanase (Manikandan et al., 2006; Shirai et al., 2007; Mamo et al., 2009; Deng et al., 2011). However, the mechanisms of esterase alkaline adaptation have barely been studied. Based on the sequence and structural features comparison (**Table 2** and **Figure 6A**), a high ratio of negatively to positively charged residues (1.64 for E69 and 1.10–1.58 for the others), a large hydrophobic surface area (2635 Å^2^ for E69 and 1516–2294 Å^2^ for the others), and a highly negatively charged surface may play roles in the alkaline adaptation of E69.

Many family IV esterases have been found to possess high thermoactivity and thermostability (Hotta et al., 2002; Li et al., 2014, 2015; Huang et al., 2016). The thermophilic feature of E69 was consistent with that of other reported family IV members (**Figure 3C**). In addition, E69 is resistant to NaCl and several organic solvents, including acetonitrile, ethanol, DMF, glycerol, and isopropanol (**Figure 4**). Although E69 exhibits a narrow substrate specificity and low hydrolysis activities toward *p*-NP esters compared with its alkaliphilic counterparts of family IV, its thermostability, halotolerant, and organic solvent-tolerant properties make E69 a good candidate for biocatalytic processes requiring high temperature, high salinity, organic solvents, and low water activity environments (Fuciños et al., 2012).

And we consider E69 a novel family IV marine esterase based on sequence and enzyme characteristics, although thermostable, alkaliphilic, and halotolerant were common marine esterase properties. First, the identities of amino acid sequences between E69 and function-characterized family IV proteins were pretty low (≤42%). Secondly, E69 has the highest optimum pH of all the reported family IV esterases. Thirdly, due to its marine origin, E69 exhibited halotolerant and organic solvent-tolerant properties, which also make it different to terrestrial original homologous esterases.

## Conclusion

The novel esterase gene *e69* was cloned from a marine isolate, *E. seohaensis* SW-135, and expressed and characterized. E69 is a halotolerant thermoalkaliphilic esterase. It exhibits some sequence and structural features that might be responsible for its alkaline adaptation, such as a high ratio of negatively to positively charged residues, a large hydrophobic surface area, and a negative surface electrostatic potential. This enzyme showed some degree of tolerance toward Triton X-100 and several organic solvents. All of these characteristics make E69 a good candidate for industrial applications.

## Author Contributions

Y-YH, ZR, S-LJ, and C-DX performed all the experiments. Y-YH analyzed the data. Y-YH, X-WX, and JL wrote the manuscript. X-WX conceived and designed the study.

## Conflict of Interest Statement

The authors declare that the research was conducted in the absence of any commercial or financial relationships that could be construed as a potential conflict of interest.

## Footnotes

1 https://blast.ncbi.nlm.nih.gov

2 https://web.expasy.org/protparam/

3 http://www.sbg.bio.ic.ac.uk/phyre2/

4 http://pymol.sourceforge.net
