# Supplementary material for: A Novel Halotolerant Thermoalkaliphilic Esterase from Marine Bacterium Erythrobacter seohaensis SW-135
Source: Front Microbiol. 2017 Nov 22;8:2315. doi: 10.3389/fmicb.2017.02315 (PMC5702849; doi:10.3389/fmicb.2017.02315)

Supplementary Material

A Novel Halotolerant Thermoalkaliphilic Esterase from Marine Bacterium *Erythrobacter seohaensis* SW135

Ying-Yi Huo, Zhen Rong, Shu-Ling Jian, Cao-Di Xu, Jixi Li and Xue-Wei Xu*

*** Correspondence:** Xue-Wei Xu: xuxw@sio.org.cn

Supplementary Figure 1. SDS-PAGE of purified E69. Lane 1, co-expressed His-tagged SUMO and E69 after cleavage by ULP1; Lane 2, purified E69 that was not captured by the Ni Sepharose column; Lane 3, the result of washing with 20 mM imidazole buffer; Lane 4, the His-tagged SUMO washed with 250 mM imidazole buffer; Lane M, marker.

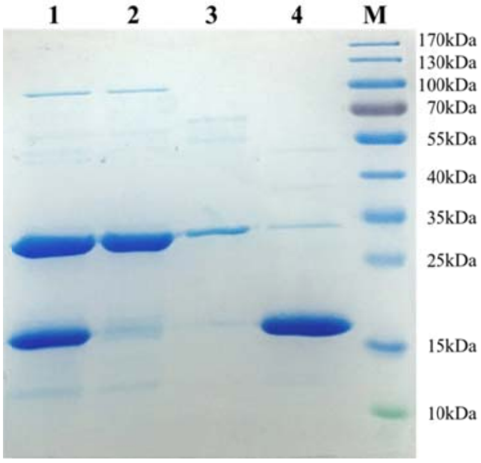

Supplement: Supplementary file 1 [file Image_1.PDF]
